# Role of protected areas for a colonial-breeding waterbird in a fragmented landscape throughout its annual cycle

**DOI:** 10.1007/s10980-024-02017-5

**Published:** 2024-12-21

**Authors:** Hugo R. S. Ferreira, José A. Alves, Frédéric Jiguet, Olivier Duriez, Thomas Blanchon, Tamar Lok, Jocelyn Champagnon

**Affiliations:** 1 Department of Biologia and CESAM – Centre for Environmental and Marine Studies, Universidade de Aveiro, Campus de Santiago, 3810-193 Aveiro, Portugal; 2 Tour du Valat, Research Institute for the Conservation of Mediterranean Wetlands, Le Sambuc, 13200 Arles, France; 3 South Iceland Research Centre, University of Iceland, Lindarbraut 4, 840 Laugarvatn, Iceland; 4 Centre d’Ecologie et des Sciences de la Conservation, UMR7204, MNHN CNRS Sorbonne Université, 43 Rue Buffon, CP 135, 75005 Paris, France; 5 CEFE, CNRS, EPHE, IRD, Univ Montpellier, 1919 Route de Mende, 34293 Montpellier, France; 6 NIOZ Royal Netherlands Institute for Sea Research, Department of Coastal Systems, PO Box 59, 1790 AB Den Burg Texel, The Netherlands

**Keywords:** *Platalea leucorodia*, Areas of strong protection, Hunting pressure, Nature reserves, Water management, Tracking data

## Abstract

**Context:**

Throughout their annual cycle and life stages, animals depend on a variety of habitats to meet their vital needs. However, habitat loss, degradation, and fragmentation are making it increasingly difficult for mobile species such as birds to find suitable habitats. Wetlands are highly productive systems of great importance to many animals, but their continued degradation threatens their capacity to support different species, including waterbirds. In this context, waterbirds are likely to benefit not only from the creation and management of protected wetlands, but also from the existence of anthropogenic wetlands, managed for economic or recreational activities.

**Objectives:**

We investigated the habitat use of Eurasian spoonbills within an extensive and heterogeneous area in Southern France, and how it varies across the annual cycle and for different age classes.

**Methods:**

We tracked 91 spoonbills of different ages throughout their annual cycle and tested for overall differences in the use of strongly protected areas in Camargue between periods and age classes. Additionally, we identified the main sites used and their management practices.

**Results:**

Our study shows that privately managed wetland areas play a complementary role to strongly protected areas: they may provide spoonbills (and other waterbirds) with suitable foraging habitat at certain periods of the year when these are less available in strongly protected areas.

**Conclusions:**

This study illustrates how the spoonbill, a moderately specialized species, is benefiting from current global changes due to its ability to use suitable habitats, natural and artificial, in fragmented landscapes. Nevertheless, reliance on privately managed wetland areas may have serious consequences for species that are highly dependent on them, and thus, habitat management promoting natural conditions may be crucial to maintain species resilience. It is therefore essential to understand how specific management actions may affect waterbird presence and habitat use, not only to enhance the effectiveness of conservation efforts, but also to promote wetland connectivity and species resilience, particularly in fragmented landscapes.

**Supplementary Information:**

The online version contains supplementary material available at 10.1007/s10980-024-02017-5.

## Introduction

Animals often rely on different habitats to fulfil their vital needs (e.g., breed, feed, moult, and shelter). However, habitat requirements for specific functions are highly variable according to species biology and often change throughout the annual cycle and lifetime (Pope et al. 2000; Johst et al. 2001). Due to habitat loss, degradation and fragmentation, the ability to find areas with suitable habitats is increasingly hard even for highly mobile species like birds (Freemark and Merriam 1986). The designation, management, and restoration of protected habitats and areas are therefore considered essential for the sustainability of highly mobile species in these patchy and complex landscapes (Geldmann et al. 2019; Leberger et al. 2020).

Wetlands are one of the most degraded habitats worldwide, with an estimated loss of 87% of their surface area since the 1700s, displaying rates that supersede forest loss threefold (Convention on Wetlands et al. 2021). Wetlands are also highly productive systems with great importance for many animals throughout their annual cycle (MEA 2005; Keddy 2010). However, their severe and continuous degradation compromises not only their capacity to host different species (including waterbirds), but also the ecosystem services they provide (Okes et al. 2008; Platteeuw et al. 2010). Surprisingly, despite this negative scenario, several waterbird species in Western Europe have been expanding and recolonizing their historical distribution range in recent decades (e.g., greater flamingo, *Phoenicopterus roseus* – Johnson and Cézilly 2009; Dalmatian pelican, *Pelecanus crispus* – Catsadorakis et al. 2015; glossy ibis, *Plegadis falcinellus* – Santoro et al. 2019; herons – Fasola et al. 2023)*.*

Some species are known to have benefited from the creation of protected areas in wetlands, such as **w**hooper swans (*Cygnus cygnus*), which have a higher survival rate inside protected areas than outside (Soriano-Redondo et al. 2023). Similarly, tracking of dabbling ducks in California revealed rapid adjustment to disturbance through increased use of protected areas (McDuie et al. 2021). Yet, some species are likely to benefit from additional actions besides the creation of protected areas, such as strengthened environmental legislation (Wetlands International 2016; Rodrigues et al. 2023). For instance, in addition to measures to mitigate colony disturbance from anthropogenic influence (e.g., aircrafts) and predators (e.g., **y**ellow-legged gulls, *Larus michahellis*), breeding flamingos have benefited from several targeted management and conservation actions to counter erosion (i.e., caused by sea level rise) and the lack of suitable nesting islands (Johnson and Cézilly 2009). Furthermore, besides the different conservation measures, some species may have benefited indirectly from other measures or factors, such as new food sources (e.g., proliferation of the red-swamp crayfish – *Procambarus clarkii*, Correia 2001; Poulin et al. 2007; garbage dumps, Plaza and Lambertucci 2017). Likewise, the existence of anthropogenic wetlands managed for economic or recreational activities (e.g., agriculture and hunting) can provide food for some waterbird species (Johst et al. 2001; Alonso et al. 2008; Hamza et al. 2015). For instance, in regions of intensive rice cultivation, rice fields provide a suitable habitat for waterbirds at different points of their annual cycle (Fasola 1996; Alves et al. 2010). In the case of hunting areas during non-breeding period, managers artificially control water levels to promote aquatic plant productivity and create ecological niches for waterfowl prey, such as seeds, small fish, and invertebrates (Tamisier et al. 1995; Isola et al. 2000). Thus, to further promote recovery and resilience of wetland-dependent birds, it is important to gain a detailed understanding of their habitat use in areas that offer multiple aquatic habitats under varying management regimes.

While habitat selection and use by highly mobile bird species has been extensively studied (Fuller 2012), studies were until recently limited by biased observational efforts in space and time (Brown et al. 2013). The emergence of tracking and other biologging technologies, such as GPS tags (Wilmers et al. 2015), allows not only to collect highly detailed information on habitat use, but also to get additional understanding of habitat functional roles, i.e. where main activities such as foraging or roosting are carried out (Ewing et al. 2018; Rodrigues et al. 2023). It is therefore currently possible to identify species needs in terms of specific habitats, understand how areas under different management regimes or protection levels are used, and use this information to develop and test the efficiency of targeted management objectives (DeFries et al. 2007; Allen and Singh 2016; McDuie et al. 2021).

The Eurasian spoonbill (*Platalea leucorodia*, hereafter spoonbill) is a long-lived migratory waterbird, distributed from the East-Atlantic Coast to Southeast Asia (Triplet et al. 2008). In Europe, spoonbills have three distinct flyway meta-populations (Champagnon et al. 2019b). Currently, the East Atlantic Flyway meta-population is steadily increasing in several breeding sites, in contrast to the Central European and Southeastern European flyways meta-populations, which are experiencing stable and declining trends, respectively (Champagnon and Kralj 2023). Spoonbills are dependent on shallow water to feed, where they tactilely forage on small fish and invertebrates, sweeping their sensitive bills from side to side through the water column (Cramp and Simmons 1977). Since 1998, spoonbills have been breeding in Camargue (Blanchon et al. 2019), and the population has increased to more than 400 pairs in 2021 (Champagnon and Kralj 2023).

Camargue is a wetland of international importance, according to the Ramsar Convention, that benefits from protection measures in its entire geographical area through overlapping international, national and regional protection statuses (Vallecillo et al. 2023), resulting in spatial variation in the level of protection, i.e. moderate or strong (according to international land protection and management categories; see methods). In general, spoonbills in Camargue are site faithful, breeding in areas that, although not strongly protected according to our classification, are relatively undisturbed (Ferreira et al. 2024). Furthermore, although areas of strong protection tend to be highly undisturbed, they may not hold abundant food resources in comparison with privately managed agricultural fields and wetlands (Johst et al. 2001; Hamza et al. 2015), particularly when management in protected areas prioritizes other taxa (e.g., Mediterranean grasslands habitat or Odonata community) rather than food availability for waterbirds. Currently, a growing number of resident spoonbills, ca. 250 individuals in recent years (Moussy et al. 2023), are also wintering in Camargue, which highlights the year-round importance of this wetland for the species. Thus, assessing how spoonbills thrive in this region with multiple land uses and habitats that provides a unique opportunity to gain a detailed understanding of the functional habitat use across multiple life stages of this waterbird species.

In order to investigate habitat use by spoonbills of different age classes, throughout the annual cycle and across an extensive and heterogeneous landscape, we fitted 96 birds with GPS tags and tracked their movements between 2016 and 2023 in Camargue. We first (i) assessed the general relevance of areas with different protection levels in Camargue for the spoonbill population. We predict a lower proportion of birds in areas of strong protection, as food abundance may be higher in areas of moderate protection, which are managed to provide space and food resources for the influx of migrating and wintering waterfowl (Brochet et al. 2009; Mathevet and Guillemain 2016). Whilst in areas of strong protection no management targeting food resources is executed; We then (ii) explored how the use of strongly protected areas changes seasonally throughout the annual cycle (i.e., breeding *vs.* early dispersal *vs*. late dispersal *vs*. wintering; see methods for the definition of each period). We predict that during breeding, spoonbills will spend considerably more time in the colonies, which are located outside strongly protected areas. Furthermore, due to the occurrence of hunting disturbance in moderately protected areas (Article 7, European Commission 2008) during the late dispersal and wintering periods, we also predict a higher use of strongly protected areas during these periods. Subsequently, (iii) we investigated the importance of strongly protected areas for different age classes (juvenile *vs*. immature *vs*. adult). Due to their accumulated experience of using the Camargue landscape and associated levels of disturbance, we predict that strongly protected areas will be mainly used by adults. Additionally, due to their higher exploratory behaviour, competition, and inexperience in foraging, immature birds often forage across larger areas (Votier et al. 2011), and consequently will likely display a dispersive behaviour and higher use of less (i.e. moderately) protected areas. Finally, (iv) we presented spatially, and temporarily explicit information of protected area use across periods for the three age classes. This showed which areas were used by spoonbills and when, allowing the presence of spoonbills in these areas to be indirectly linked to specific management actions.

## Material and methods

### Study area and population

**Fig. 1:**
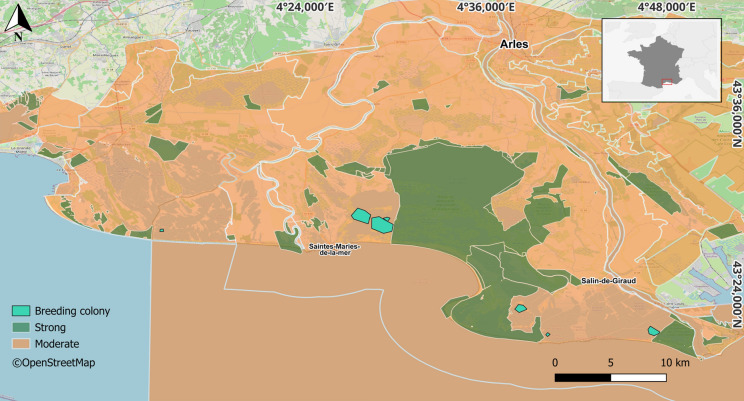
Map of the Camargue region along the Mediterranean coast of France, highlighting areas of moderate (orange) and of strong protection (dark green). Areas were classified based on the IUCN categories stated in Table 1. The main breeding colonies in Camargue where GPS tags were deployed are shown (light green) and are all located within areas of moderate protection. White lines are the boundaries of the areas described in Table 1

Camargue extends over an area of 180,000 ha and comprises a diverse mosaic of habitats, including natural and semi-natural wetlands, two major salt pans, rice fields, and other agricultural areas (Galewski and Devictor 2016). It encompasses a complex diversity of management techniques and stakeholders, involved in the administration of areas under different legal regimens (e.g., national, private, and regional reserves) and different land uses (e.g., farming, salt pans, and waterfowl hunting areas; Galewski and Devictor 2016). The existing types of protection are diverse and overlapping, ranging from conventional and regulatory protection to protection through land ownership, and compliance to European or international laws and conventions (see Table 1 and Fig. 1 for further details and a breakdown of the types of protection). Hereafter, in order to standardize the different types of protection, we consider all the areas that contain one of the first four categories of the International Union for Conservation of Nature protected area classification (IUCN 1994) to belong to strongly protected areas, encompassing 26% of the study area. These areas are strictly managed as nature reserves, with no waterfowl hunting activities and minimal disturbance, while in other areas recreational and economic activities are generally allowed and often intensive.

**Table 1:** Protected areas included in the study area according to Lefebvre and Moncorps (2010)

Protected area classification	IUCN categories	Protection type	Level of protection	Surface area (ca. ha)
Camargue National Nature Reserve	I–IV	A	Strong	14,127
Vigueirat National Nature Reserve	I–IV	A	Strong	1,200
*“Site classé”*^(1)^	III	A	Strong	14,150
Arrêté de protection de biotope^(2)^	IV	A	Strong	788
Mahistre & Musette Regional Nature Reserve	IV	A	Strong	263
Scamandre Regional Nature Reserve	IV	A	Strong	148
Tour du Valat Regional Nature Reserve	IV	A	Strong	1,865
*“Conservatoire du Littoral”* dependencies ^(3)^	IV–V	B	Strong	29,497
Camargue Regional Nature Park	V	C	Moderate	99,849
*Espaces naturels sensibles* ^(4)^	V	B	Moderate	3,851
*“Sites inscrits”* ^(2)^	V–VI	A	Moderate	108,678
Camargue Ramsar Site	^(6)^	D	Moderate	126,288
Camargue/Delta du Rhône Biosphere Reserve	_(6)_	D	Moderate	193,021
Special Protection Areas (Natura 2000)^(5)^	_(6)_	D	Moderate	243,187
Special Areas of Conservation (Natura 2000)^(5)^	_(6)_	D	Moderate	163,502

Type of protection: A—statutory; B—property-based; C—conventional; D—under a European or international law. The level of protection corresponds to the category considered for this study: moderate or strong

(1) Protection under the laws of 21 April 1906 and 2 May 1930 (articles L. 341-1 to 22 of the French Environment Code)

(2) Protection system created by decree no. 77-1295 of 25 November 1977 (codified in the French Environment Code in articles L. 411-1, L. 411-2, R. 411-15, R.411-16 and R.411-17)

(3) Name of the areas included: Bois de Tourtoulen; Camargue Gardoise; Domaine de Rousty; Embouchure du Petit-Rhône; Étangs et Marais des Salins de Camargue; La Palissade; Domaine des Grandes Cabanes du Vaccarès; Marais du Vigueirat; Sainte-Cécile; Vaccarès

(4) Name of the areas included: “Étangs des Impériaux; Malagroy & Consecanières”; “Domaine du Ménage”; “Château d’Avignon”; “Grandes Cabanes du Vaccarès”; “Clos de la Royalette”

(5) SPA & SAC Natura 2000 sites are respectively designated under the Birds and the Habitats Directives

(6) IUCN category not attributable due to the absence of a defined legal status in France (Ramsar, Biosphere reserves) or because management is too heterogeneous (Natura 2000)

A total of 94 chicks (Table S1 and Fig. S1) were captured in the colonies during the breeding seasons of 2016–2023 (Table S1 and Fig. S1). Chicks were captured by hand during their pre-fledging period (ca. 28–33 days) and individually fitted with solar-powered GPS/accelerometer GSM tags (Druid, www.druid.tech; Ecotone, www.ecotone-telemetry.com; Ornitela, www.ornitela.com). Two additional adults were captured using a noose trap deployed at the nest and subsequently tagged, thus increasing the number of individuals considered in this study to 96. All the tags (except two that were attached directly to the leg of the chicks) were attached using a Teflon ribbon backpack harness (Thaxter et al. 2014). PVC engraved rings were also attached to the leg, for visual individual identification from a distance. The body mass of the chicks was recorded using a Pesola spring balance (± 10 g). The total combined weight of the tags (25 g), harness, and PVC ring was ca. 36 g*,* thus below the 3% threshold (Phillips et al. 2003) of the body mass of the tagged spoonbills (mean ± SD = 1625 ± 186 g*, range* = 1200–2120 g, *n* = 95; one individual was not weighed, see Table S1 for details). During the capture and GPS fitting procedures, birds were handled with the utmost care by qualified and trained ringers validated by the French national ringing scheme (CRBPO, Programmes personnels PP580 and PP1190 MigraLion).

### Data collection and selection

Tags were programmed to record a GPS fix every 10 min whenever possible (with an associated error of ≤ 6 m) but for battery saving the fix frequency could decrease to one every six hours. The actual fix frequency averaged for Druid and Ornitela tags was 14.3 min ± 1.6 SE, and for Ecotone tags 408.8 min ± 77.6 SE. Data were filtered to include only GPS fixes within the Camargue study area (from 43°19’N to 43°42’N and from 4°06’E to 4°54'′E; Fig. 1), resulting in the exclusion of four individuals with no GPS fixes recorded in Camargue. To reduce uncertainty, only GPS fixes that used at least four satellites for geo-positioning were retained. In the case of four individuals (Table S1: APVA, ATTL, FBZA and FBZB), no information on the number of satellites used was available so all data were included. In order to exclude their movement inside the colony before fledging, we only considered the GPS fixes of juveniles after they had spent a complete day outside the breeding colony. For the two adults, all fixes recorded after tagging were considered. The last recorded GPS fix corresponds either to the final tracked day of this study (May 15th, 2024; *n* = 30 birds still alive and actively being tracked), a mortality event (*n* = 36), tag failure (*n* = 3) or because individuals moved into an area of poor GSM coverage, hence their fate is unknown (*n* = 27).

Spoonbills in Camargue breed over a relatively long period with egg-laying occurring between February and June (Blanchon et al. 2019). We considered the start of the breeding period as the 15th of February. Each individual was categorised in age classes according to the date of each GPS fix: (i) juvenile, all fixes from the date of its fledging to the start of the next breeding period; (ii) immature, all the fixes between the start of the second breeding period and the start of the fourth one; (iii) adult, all fixes after the start of the fourth breeding period for the birds tagged as chicks and all fixes for the two individuals tagged as adults. Although spoonbills have delayed maturity and are considered to recruit in their fourth calendar year (Cramp and Simmons 1977), a low proportion of breeders can breed at two years old (third calendar year; Champagnon et al. 2019a). Three of the tracked individuals were seen at the colony and displaying breeding behaviour during their third calendar year, so for this study we considered individuals to be adults (breeders) at the start of their fourth breeding period. In total, 89 different juveniles were considered for this study; 19 of those birds also provided data as immatures; and seven of those further provided data as adults, thus totalling nine adults with relevant data. For further details on the number of spoonbills fitted with tags per year and on which individuals provided information at each age class, see Table S1 and Fig. S1.

In order to consider the seasonal variation in use of protected areas due to different ecological requirements (e.g., breeding, pre-migratory fuelling) and to the changes in local conditions throughout the year (specifically in management, climate, and hunting season—21 August 31 January), we divided the year into four periods: (i) breeding (15 February–14 May), when breeding adults are mainly present at the colonies and only a few juveniles may have fledge in some years; (ii) early dispersal (15 May–14 August), when many juveniles have fledged and begin to disperse, while late breeding adults (or those with a second clutch) remain in the colonies; (iii) late dispersal (15 August–14 November), when colonies are generally empty after fledging of juveniles and migratory birds of all age classes gradually leave Camargue to reach other locations; and (iv) wintering (15 November–14 February), when only resident birds are present in the Camargue. Although spoonbills usually remain on the wintering grounds until reaching breeding maturity, some immature birds do return to the breeding area, where they likely have exploratory behaviour without breeding (Lok et al. 2015; Tenan et al. 2017).

### GPS data treatment

Areas highly revisited by animals are often considered to be of ecological significance and their identification can provide important insights into the life history of populations (Bracis et al. 2018). To identify fixes registered by the spoonbills when they are potentially breeding, resting, and foraging, causing multiple revisits of the same area, we conducted a revisitation analysis using the *recurse* package (Bracis et al. 2018). This analysis allowed us to calculate the number of times (revisits) an individual trajectory entered a circular area centred on each position of the trajectory. To perform this calculation, we defined a circular radius of 100 m, obtained by following an adapted procedure of the exploratory analysis performed by Rodrigues et al. (2023) (for details, see Table S3; Fig. S2; and Fig. S3). This analysis resulted in the same radius (100 m) as the one obtained by Rodrigues et al. (2023), being a good compromise between avoiding spurious results, without neglecting changes in habitat use due to overlap of GPS fixes if using a larger radius. To ensure that revisits were independent not only in space but also in time, a threshold of 30 min between two consecutive GPS fixes was defined. This means that if after a given GPS fix, the following one was recorded within a radius of 100 m during a 30-min period, it was not considered to be a new revisit. Due to computational limitations and to avoid pseudoreplication issues, only GPS fixes of the same aggregated group (age class; land protection level; period; and year) were considered as potential revisits. Hereafter, only the GPS fixes with the most revisited circles (> 5% quantile of each individual) were kept (*n *_*GPS fixes*_ = 1,628,448; *n *_*Ind*_ = 92). Finally, to avoid revisit misclassifications we kept only fixes which were revisited at least three times (*n *_*GPS fixes*_ = 1,565,015; *n *_*Ind*_ = 92; Table S4). When inspecting the data, one individual (ring AZZT) had only one valid GPS fix revisited several times and thus was removed from the analysis. Furthermore, two juveniles (rings A22C and ATTL) only had respectively three and one valid fixes registered during the breeding period and thus, their fixes for this period were removed due to their small number of data. The dataset analysed totalised *n *_*Revisitation fixes*_ = 1,565,010 and *n *_*Ind*_ = 91 (Table S4).

### Data visualization—heat maps & day plots

To evaluate the importance of specific areas with varying levels of protection across the annual and life cycles, we produced spatially explicit heat maps of spoonbills GPS fixes for each combination of age class and period. We successfully identified the most relevant areas for each type of land protection level and proceed to discuss how their management could affect their use by spoonbills. Furthermore, the percentage of GPS fixes for each age class and period of the year was calculated. To identify temporal peaks in the use of areas with strong protection, we plotted the average proportion of GPS fixes located in areas of strong protection per Julian day and for each age class.

### Statistical analysis

To test the relationship between the number of GPS fixes (i.e. those revisited ≥ three times, henceforward revisitation fixes) and the variables of interest, we used generalized linear mixed models in an information-theoretic model selection framework (Burnham and Anderson 2003). To account for overdispersion, we considered a negative binomial distribution (*Ml* and *nlminb* optimizer; *glmmTMB* package **–** Brooks et al. 2017) and manually developed models for competition. We considered the total number of revisitation fixes as a response variable and land protection level (*Protection*, categorical with two levels), period of the year (*Period*, categorical with four levels), and age class (*Age*, categorical with three levels) as fixed effects. To incorporate the dependency among revisitation fixes from the same individual (*Ind*) and colony of birth (*Colony*, categorical with seven levels), we used *Ind* nested within *Colony* as a random intercept. Additionally, to incorporate the dependency among observations of the same year, year was also considered as a random intercept (*Year*, categorical with eight levels). No interaction between *Age* and *Period* was considered due to convergence issues likely caused by the absence of data from juveniles during the breeding period. Using the *DHARMa* package, we performed a Dharma nonparametric dispersion test which confirmed a lack of overdispersion (overdispersion = *0.47*; *p* = *0.25*). Consequently, we obtained the following equation as the initial complex model:

1 $$\begin{aligned} \log \left( {Fixes_{\mu ijkl} } \right) = & Age_{ij } \times Protection_{ij} + Protection_{ij } \times Period_{ij} \\ & + Colony_{i} \times Ind_{ij} + Year_{i} \\ & Colony_{i} \sim N\left( {0,\sigma_{Colony}^{2} } \right) \\ & Ind_{ij} \sim N\left( {0,\sigma_{Ind}^{2} } \right) \\ & Year_{i} \sim N\left( {0,\sigma_{Year}^{2} } \right) \\ \end{aligned}$$

The different models were ranked and selected according to their Akaike´s Information Criterion adjusted for small sample size (*AIC*_*c*_*;* Anderson and Burnham 2002). In order to explore the differences among the marginal means of each group, subsequent pairwise Tukey HSD post-hoc test comparisons were conducted to the most parsimonious model using the *emmeans* package (Lenth et al. 2024). A Sidak correction was applied, which adjusts for the family-wise error rate across all tests and provides a more conservative control over Type I errors (Sidak 1967).

All analyses were carried out using R version 4.2.1 (R Core Team 2022) and plots were created using the packages *ggplot2* (Wickham 2016) and *sjPlot* (Lüdecke et al. 2023)*.* To perform additional spatial data analysis and the visualization of the geographical data, QGIS, version 3.34, was used.

## Results

**Fig. 2:**
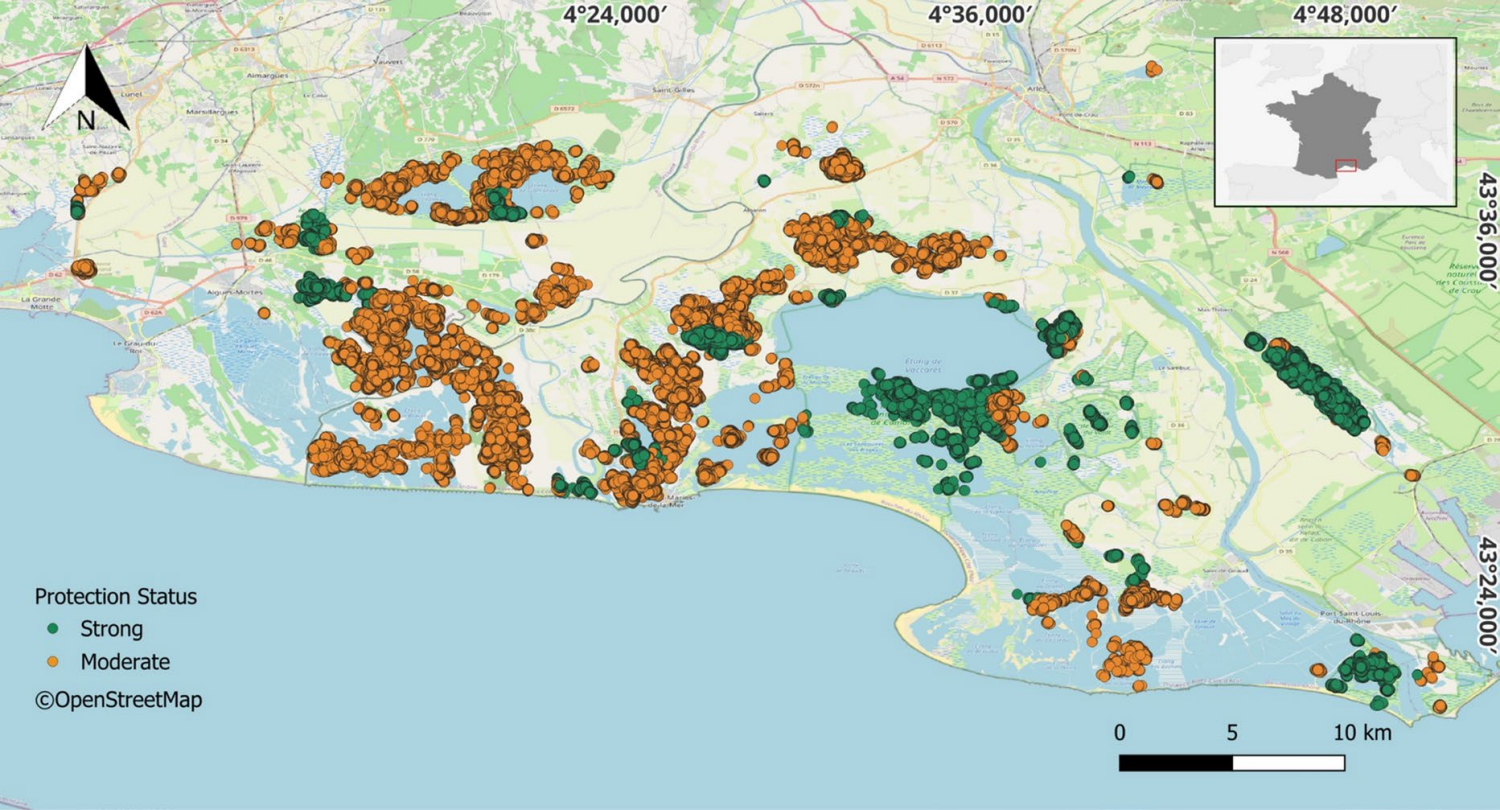
Distribution of revisitation fixes (*n *_*Revisitation fixes*_ = 1,565,010; *n *_*Ind*_ = 91) across the study area classified according to protection levels: moderate (orange) and strong (green)

From the 91 tagged spoonbills used in the analysis (Fig. 2), 82 individuals produced revisitation fixes in strongly protected areas. These fixes represented on average 36% (± 5 SE) of the total amount of revisitation fixes of individuals. The proportion of fixes located in strongly protected areas varied widely between individuals, from zero to 100%. Four individuals showed values exceeding 90% (± 2 SE), nine individuals less than 10% (± 4 SE), and nine with zero fixes in strongly protected areas.

When considering the variation in the amount of revisitation fixes per individual (Eq. 1), the most complex model was also the most-supported model (Model 13; Table 2).

**Table 2:** Generalized linear mixed models’ selection for amount of revisitation fixes (*n*_*Revisitation fixes*_ = 1,565,010; *n *_*In*d_ = 91)

Number	Model	*df*	AIC_c_	ΔAIC_c_	Akaike weight
**13**	**Age × Protection + Period × Protection**	16	10,309.7	0.0	0.8
12	Age + Period × Protection	14	10,312.6	2.9	0.2
9	Age × Protection	10	10,323.0	13.2	0.0
11	Period × Protection	12	10,328.9	19.2	0.0
10	Age × Protection + Period	13	10,336.5	26.8	0.0
8	Period + Protection	9	10,345.8	36.1	0.0
6	Age + Period	10	10,350.8	41.0	0.0
7	Age + Period + Protection	11	10,354.5	44.8	0.0
5	Age + Protection	8	10,360.5	50.8	0.0
4	Period	8	10,362.8	53.1	0.0
3	Age	7	10,363.2	53.5	0.0
1	Constant	5	10,386.0	76.3	0.0
2	Protection	6	10,386.3	76.6	0.0

*Ind* was considered as a random variable nested in *Colony*. Additionally, *Year* was also considered as a random variable. Models were ranked according to AIC_c_, and the preferred model is indicated in bold

**Fig. 3:**
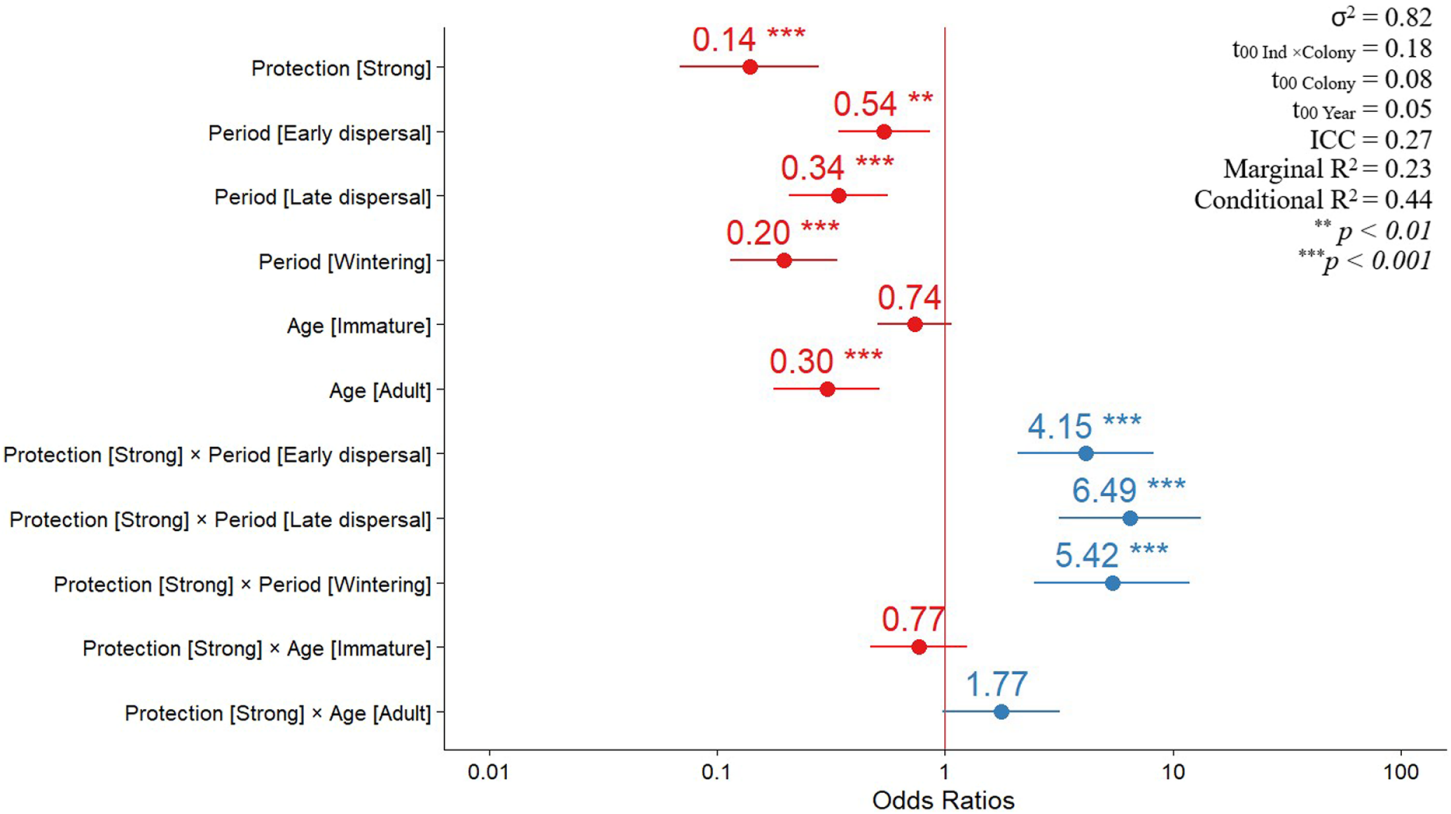
Odds ratios of a revisitation fix occurring according to: protection level (Moderate—Reference); Period (Breeding—Reference); Age (Juvenile—Reference); Protection × Period (Strong × Breeding—Reference); Protection × Age (Strong × Juvenile—Reference). Red colour represents lower odds of revisitation fix occurring when compared to the reference level, while blue colour represents higher odds. The “neutral” line (red vertical line) indicates no effect when intercepted. Horizontal lines indicate 95% confidence intervals. For details check Table S5

This model retained both interactions (*Age* × *Protection*; *Period* × *Protection*), hence there are significant differences in how protected areas were used according to the age of the individual and the period of the year (Fig. 3).

**Fig. 4:**
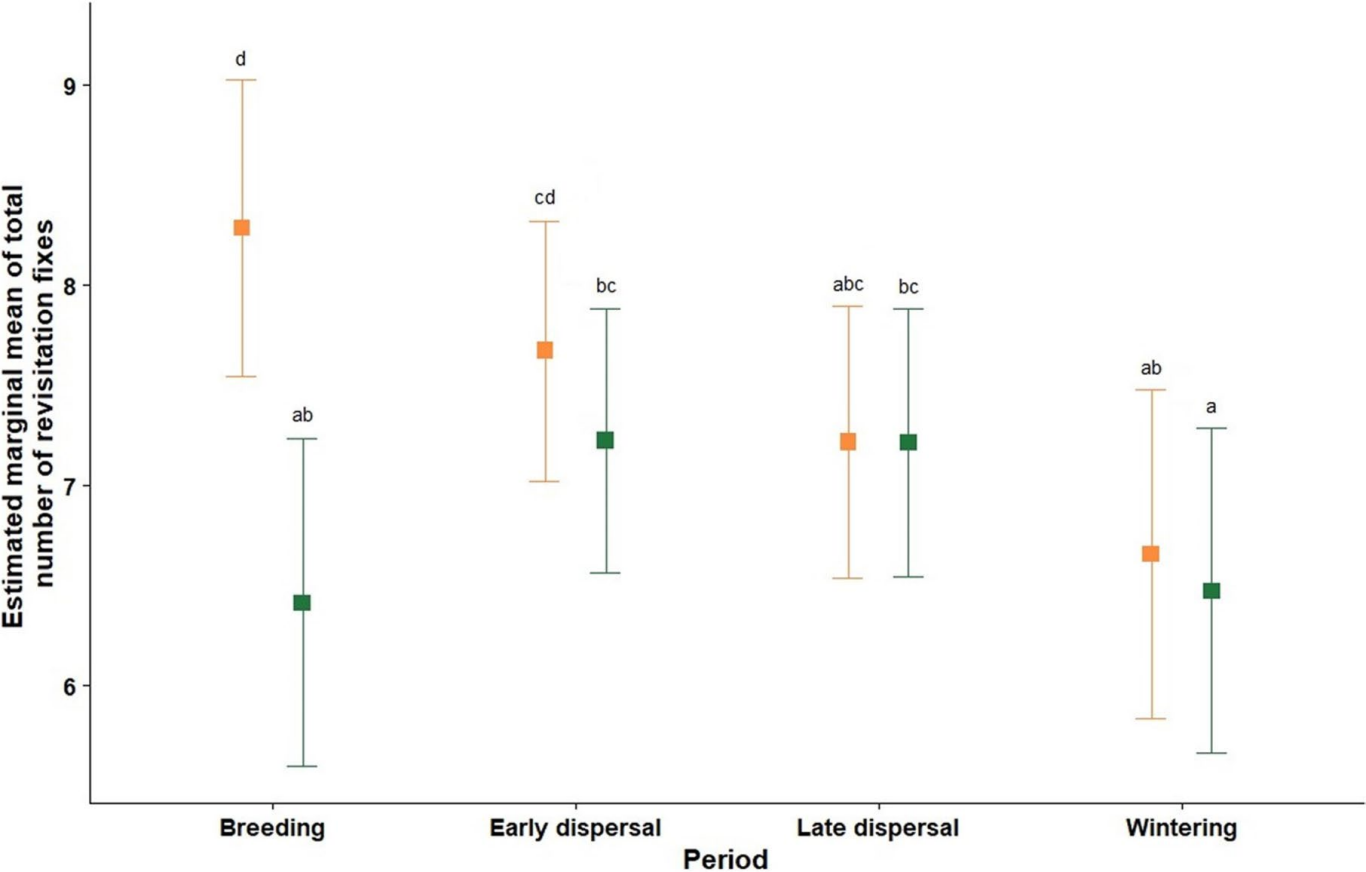
Post-hoc comparisons of estimated marginal mean of total amount of revisitation fixes between levels of protection (Moderate—orange; Strong—green) throughout the annual cycle. Vertical lines indicate 95% confidence intervals after Sidak correction. Different letters indicate statistical differences between groups (*p* < *0.05*)

When comparing the combined groups of annual period and protection level, our results indicated that there were significantly fewer revisitation fixes located within areas of strong protection during the breeding period (Fig. 4). No differences in the number of fixes between protection levels were detected in the remaining periods. Furthermore, the number of revisitation fixes located in areas of moderate protection appears to be lowest during the wintering period, despite that no difference was detected with the late dispersal period.

**Fig. 5:**
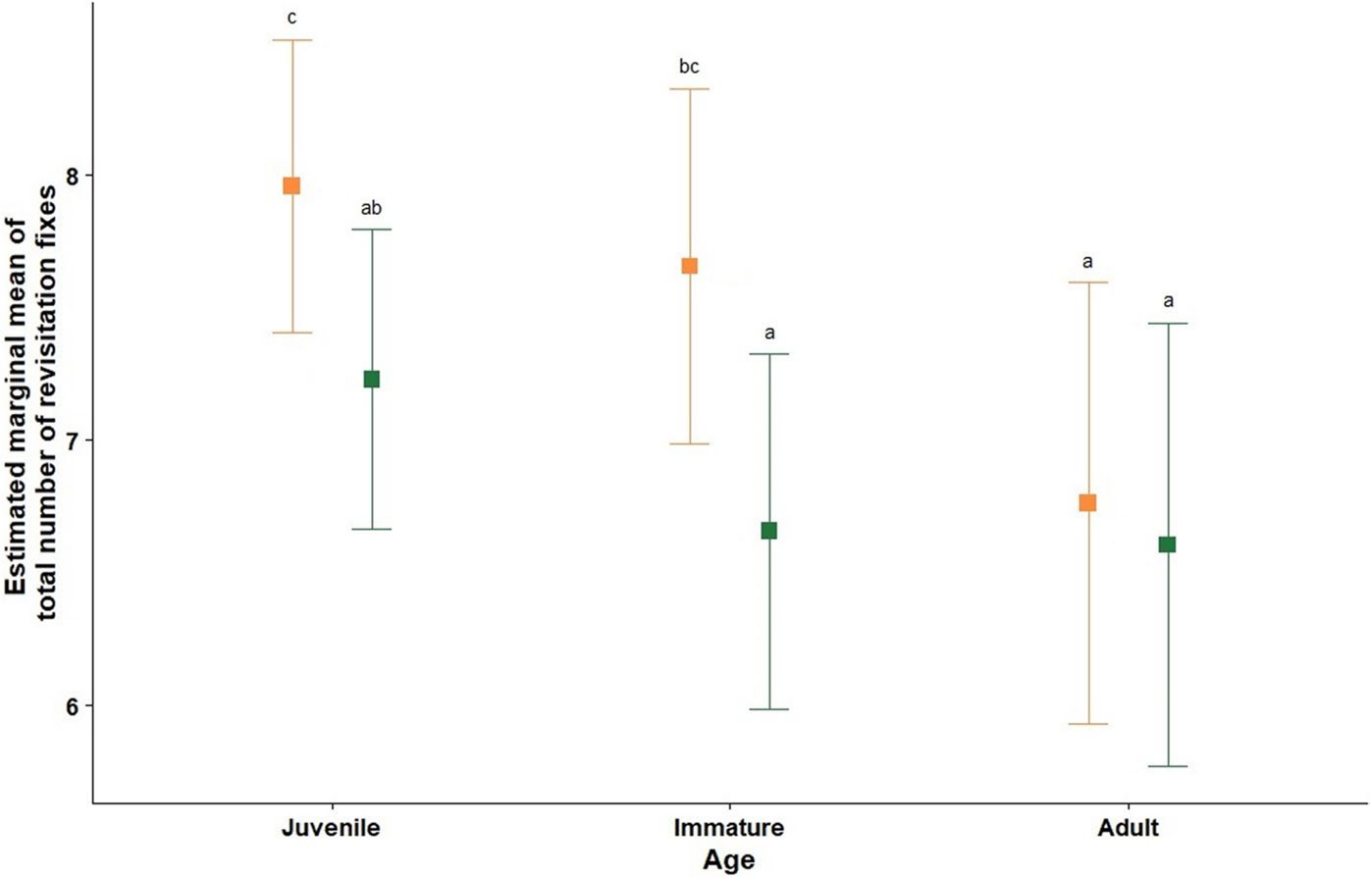
Post-hoc comparisons of estimated marginal mean of total revisitation fixes between protection levels (moderate—orange; strong—green) across the different age class. Vertical lines indicate 95% confidence after Sidak correction. Different letters indicate statistical differences between groups (*p* < *0.05*)

Juveniles and immatures had significantly more revisitation fixes within areas of moderate protection than in areas of strong protection, whereas no differences were detected for adults (Fig. 5).

**Fig. 6:**
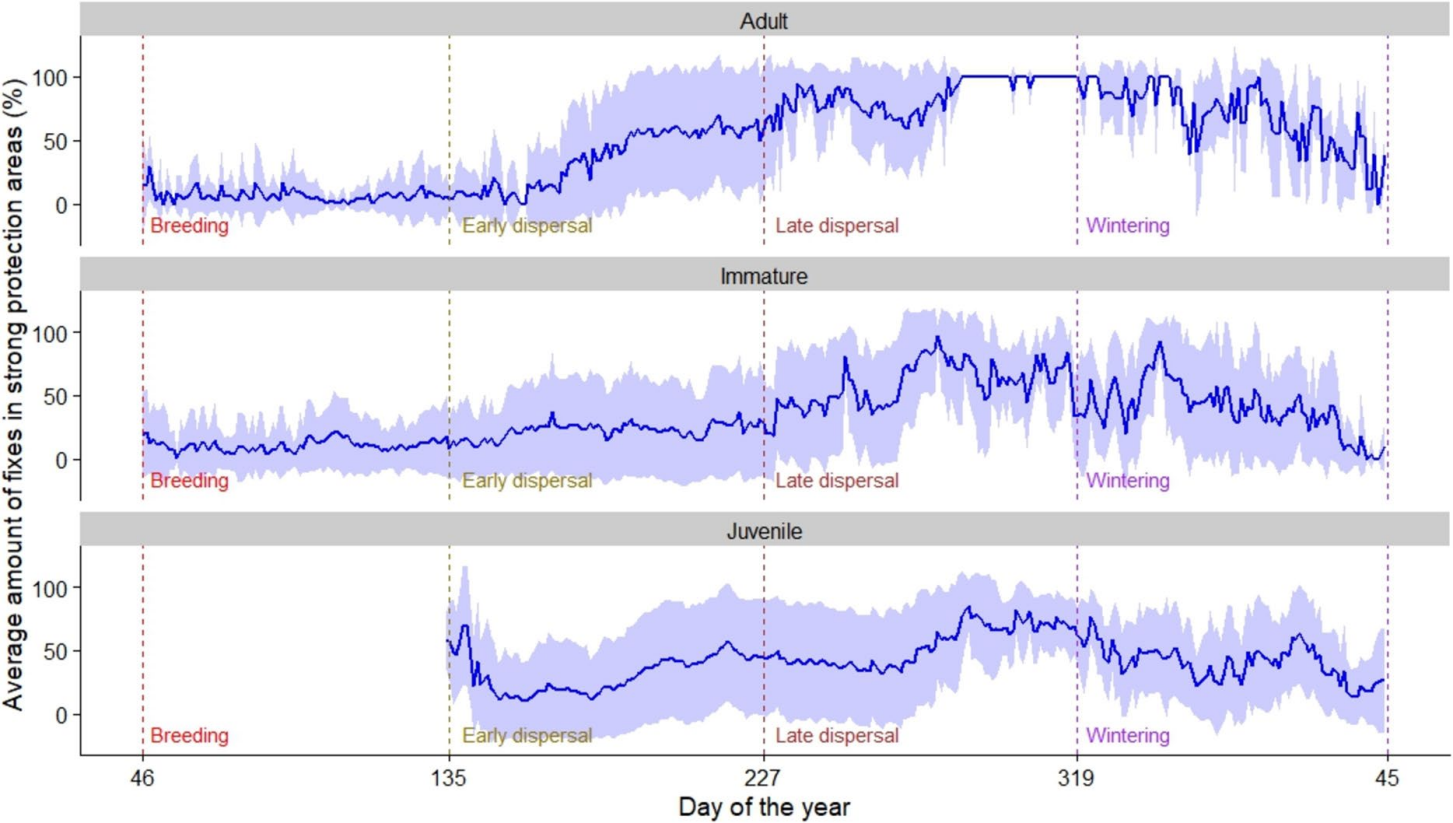
Variation in the average frequency of revisitation fixes (*n*_*Revisitation fixes*_ = 1,565,010; *n*_*Ind*_ = 91), throughout the annual cycle according to each period, for each age class (juvenile, immature, and adult). Dashed vertical lines delimitate the different periods: Breeding; Early dispersal; Late dispersal; and Wintering. No fixes during Breeding were considered for juveniles. Shaded-blue area represents standard deviation

The plot of the average proportion of revisitation fixes inside areas of strong protection (Fig. 6) shows a strong increase in the use of these areas during early dispersal for adults. Juveniles appear to decrease their use of strongly protected areas during the early wintering period, while the decrease for adults is more pronounced in the later part of this period.

**Fig. 7:**
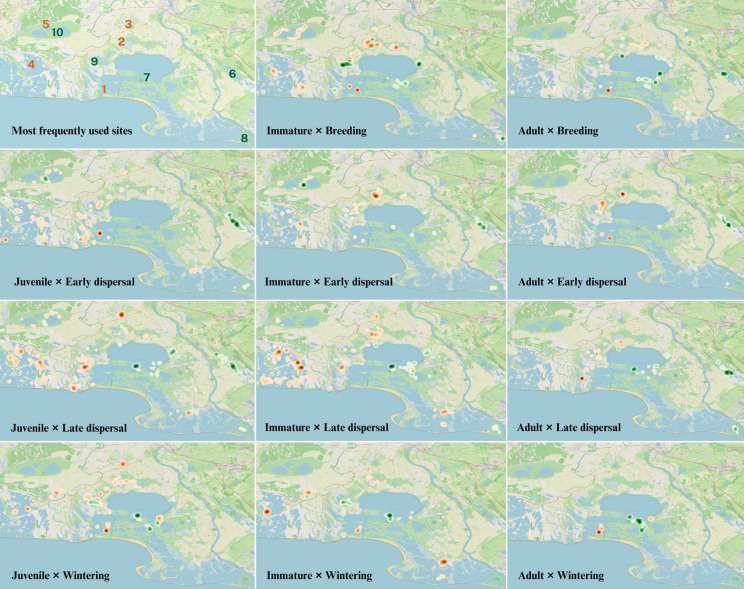
Heat maps of revisitation fixes according to protection levels (moderate—orange dots; strong—green dots), in each period of the annual cycle (rows: breeding, early dispersal, late dispersal, and wintering), and across age classes (columns: juvenile, immature, and adult). The top left map identifies the five most frequently areas across all classes of age and of protection (moderate—orange; strong—green; see text for details)

When plotting each combination of age class and period of the year (Fig. 7), we successfully identified the most relevant areas (with highest concentration of revisitation fixes) within areas of moderate (orange) and strong protection (green). In the case of the areas of moderate protection, the data highlights the following sites: 1—Sensitive Natural Area of the *Étang des Impériaux* managed by Department council; private estates managed for duck hunting (2—*Basse Méjanes*; 3—*Tamaris*; 4—*Lairan*); and communal 5—*Etang du Crey*. As for the areas of strong protection, they were identified to be all within the *“Conservatoire du Littoral”* dependencies (6—*Vigueirat* National Nature Reserve; 7—Camargue National Nature Reserve; 8—*Domaine de la Palissade* protected area; and 9—*Domaine des Grandes Cabanes du Vaccarès*), except for 10—*Scamandre* Regional Nature Reserve (see top-left panel in Fig. 7 for the location of these sites).

## Discussion

Our results indicate that spoonbills in Camargue are selective in the areas they use, with both moderately and strongly protected areas contributing to overall wetland suitability in a fragmented and highly anthropogenic landscape. This is suggested by the similar use of areas of moderate and of strong protection during the different periods of their annual cycle, apart from the breeding period, when spoonbills are highly concentrated in colonies within moderately protected areas. Indeed, GPS fixes were highly concentrated in areas managed to promote the presence of waterbirds for recreational (e.g., ecotourism and hunting activities) or for conservation purposes (highlighted in the top-left panel of Fig. 7). Overall, juveniles and immatures were significantly less likely to use areas of strong protection (Fig. 5) and appear to be more dispersive using several different areas over the annual cycle (Fig. 6, with lower percentages than adults across the annual cycle; Fig. 7). Finally, adults did not differ in their use of different types of protected areas and had a high concentration of GPS fixes in a smaller number of used areas than less experienced individuals (Fig. 7). Additionally, the areas with high concentrations of adult GPS fixes were generally the same across the different periods. This suggests that less experienced individuals might be more dispersive and use a higher number of different areas to avoid competition, or simply explore more sites because they have not yet identified the most suitable ones, as discussed in other studies (Rodrigues et al. 2023).

### Overall relevance of strongly protected areas

Despite evidence that the level of protection of protected areas is essential to determine the effectiveness of protected areas (Di Lorenzo et al. 2020; Wauchope et al. 2022), animals usually use a variety of habitats with different levels of protection (Soriano-Redondo et al. 2023). Thus, given that the entire Camargue is protected to some extent (Lefebvre and Moncorps 2010; Vallecillo et al. 2023), that several areas of moderate protection are managed to attract or protect waterbirds (Galewski and Devictor 2016), and that spoonbills are opportunistic foragers with some flexible diet (El-Hacen et al. 2014; Rodrigues et al. 2023), it is not surprising that we did not find a significant selection of strongly protected areas overall.

That moderately protected areas were significantly more selected during the breeding period is explained by the analysis having considered revisitation fixes of immatures and adults only. At this period of the annual cycle, adults spend about half of their days in the breeding colonies to incubate their eggs or attend their chicks. Immatures also spend time at the colonies, though to a lower extent, gathering (social) information, or even attempting to breed (Lok et al. 2013; Tenan et al. 2017). In Camargue, spoonbills nest on small islets to avoid terrestrial predation by Eurasian wild boar (*Sus scrofa*) and red fox (*Vulpes vulpes*) (Champagnon et al. 2021). These islets are mainly located in the *Étang des Impériaux* (site 1 in Fig. 7), an area of moderate protection (according to our classification). But this area benefits from an additional layer of protection as part of Natura 2000 and as a sensitive natural area (ENS, Table 1), actively managed by the Bouches-du-Rhône Departmental Council to avoid disturbance caused by anthropogenic activities. Considering that the other major spoonbill colonies in France (Marion 2019) are also located in areas of strong protection (according to our classification), this underlines that spoonbills, at least in Europe, require active management and strong protection measures to avoid anthropogenic disturbance of their colonies in order to improve productivity (Mikuska et al. 2023; Kazantzidis et al. 2024).

Camargue is one of the major waterfowl (primarily ducks) hunting areas in Europe (Mondain-Monval et al. 2009). Waterfowl hunting is therefore one of the main management objectives of several private wetlands in Camargue (Mondain-Monval et al. 2009; Guillemain et al. 2010; Galewski and Devictor 2016). Private areas are often actively managed to provide space and food resources for migrating and wintering waterfowl, by artificial flooding, creation of artificial ponds, management of water levels, and scattering of seeds as bait (Brochet et al. 2009; Mathevet and Guillemain 2016). On the other hand, areas under strong protection are less likely to be managed to provide abundant food resources, but rather to allow for the ecosystem to persist in its natural rhythm.

Spoonbills in Camargue are often observed feeding in private areas managed for duck hunting, such as *Basse Méjanes* (site 2 in Fig. 7), *Lairan* (site 4 in Fig. 7)*,* and *Tamaris* (site 3 in Fig. 7). As high food availability for waterfowl is the main priority for hunting managers during the hunting season (i.e., during most of the late dispersal and wintering period), the marshes in these areas can be attractive for spoonbills throughout the year. During the breeding period and before the onset of the hunting season (21st of August) these may remain permanently flooded and relatively undisturbed, providing good conditions for spoonbills. In addition, every four or five years, managers may practice the “*assec*”, i.e., drying out their estates to manage exotic vegetation (e.g., floating primrose-willow—*Ludwigia peploides*) and to prevent siltation. Some managers may even carry out this practice annually or in rotating sections in large estates. During *assec*, the receding water, when not actively drained, promotes the gathering of prey (particularly fish) in the remaining “pools”, making them easily accessible to waterbirds (Beerens et al. 2011). Subsequently, in June and July (early dispersal), private wetlands are flooded, including temporary ponds that would otherwise be dry, in order to maximise food availability (aquatic plant production) in preparation for the arrival of waterfowl in late summer (late dispersal; Tamisier and Grillas 1994; Davis et al. 2014). Even if management practices vary among the 200 hunting estates in Camargue and over years (Mondain-Monval et al. 2009), there will usually be some areas that provide good conditions to spoonbills, thus potentially explaining the high number of different moderately protected sites they use (Fig. 7).

### Seasonal change in the use of strongly protected areas

In a variety of species, strongly protected areas have been shown to be particularly important during the breeding and wintering season, when resources might be less abundant and disturbed due to hunting might be higher in moderately or non-protected areas (Gaget et al. 2021; Soriano-Redondo et al. 2023). Soriano-Redondo et al. (2023) revealed that whooper swans wintering in nature reserves have higher survival rates than the ones wintering in non-protected areas, thanks to management actions such as protection against natural predators, complete hunting prohibition, and food supply. Surprisingly, in Camargue, there was no significant increase in the use of strongly protected areas by spoonbills during the late dispersal and wintering periods, despite coinciding with the waterfowl hunting season. This could be explained by the variety of hunting practices in Camargue and by the large size of the hunting estates used by spoonbills, such as *Lairan*, a 697 ha estate where hunting takes place at varying intensities, and some part of the estate are only hunted once a month. Similarly, *Basse Méjanes* (489 ha) and *Tamaris* (16**5** ha) are relatively large estates, hunted only once a week or less. Furthermore, several private hunting areas in Camargue (ca. 57% of the hunting estates—Mondain-Monval et al. 2009) prohibit hunting in certain parts of their estates, that serve as waterfowl refuges (Vallecillo et al. 2023). Thus, waterfowl hunting activities in some private areas are unlikely to be sufficiently frequent and extensive to significantly deter spoonbill presence, except only for some periods or some locations of active hunting.

The overall average percentage of adult revisitation fixes did increase in strongly protected areas during the hunting season (from c.a. 25 to > 60%, Fig. S4). Although, this increase was not significant in our models, in terms of revisited fixes in strongly protected areas (Table S5), some strongly protected areas, such as the Camargue national nature reserve (> 14,000 ha, site 7 in Fig. 7), have a high density of revisitation fixes during the wintering period. This high revisitation is based on a single individual, but is supported by aerial surveys carried out monthly during the wintering period, that consistently identify this reserve as the main wintering area of spoonbills in Camargue (Tamisier and Dehorter 1999; Vallecillo et al. 2023). Contrary to the adults, less experienced individuals did not show such an increase in their use of strongly protected areas during the hunting season.

The number of revisitation fixes in strongly protected areas is lower for less experienced birds during the wintering period (Fig. 6), compared to adults (Fig. S5). These results, despite statistically not significant, may suggest a density-dependent process, consistent with the dominance-competitive hypothesis (Weimerskirch et al. 2014; Verhulst et al. 2014) positing that more experienced (adults) individuals push less experienced individuals into less suitable areas. Alternatively, juveniles may not yet have realised the potential benefits of using these areas during periods of higher disturbance or potentially lower food availability. The fact that disturbance may affect spoonbills (and other waterbirds) more during the wintering period than during late dispersal may be related to the increased hunting pressure that occurs during the wintering period. While hunting pressure is strong at the onset of the season especially on the released mallards (*Anas platyrhynchos*), some large privately managed areas that do not release ducks may wait for the arrival of more migratory ducks (around October and November) to carry out their activities. Which is supported by the quadratic distribution of hunting bags in the Camargue (Tamisier and Dehorter 1999). However, this could also be connected to fish availability in strongly protected areas which may increase during the winter (Bouchard et al. 2022). This increase in fish availability is also supported by the observation that fish-eating species such as great cormorants (*Phalacrocorax carbo*) and grebes (*Podicipedidae*) congregate at such sites.

### Variation in use of strongly protected areas between age classes

Besides using moderately protected areas more frequently than adults, juveniles and immatures also were more dispersive and used a higher number of areas in Camargue, as indicated by the heat maps (Fig. 7). These results could be related to exploratory behaviour typical of these age classes, or simply to the process of finding suitable areas, or to competition for the use of strongly protected areas (Rodrigues et al. 2023; Hertel et al. 2023). Juveniles and immatures are still developing their foraging skills and knowledge of the environment, and have been shown to be less competitive when large numbers of individuals congregate in the same area (Rotics et al. 2016; Votier et al. 2017). Indeed, tracked juvenile spoonbills in Ria Formosa (southern Portugal) were also found to use a considerable number of different areas (although with no formal comparison with adults), which was hypothesised to be due to opportunistic foraging behaviour and competition avoidance.

### Relationship between management and waterbird presence

To better understand which management measures might be associated with the presence of spoonbills in certain areas and at certain periods, we identified five clusters of heavily used areas for each type of protection (Fig. 7). One of them is the Vigueirat national nature reserve (site 6 in Fig. 7), a strongly protected area of 1200 ha, with actively managed water levels to provide optimal conditions for waterbird species, considering climatic conditions, the annual cycle, and the biology of the target species (e.g., ducks in winter, *Ardeidae*, terns, waders and spoonbills in spring and summer). Vigueirat exemplifies other strongly protected areas in terms of management. Here, managers maintain high-water levels until July to create favourable breeding conditions for various waterbirds (e.g., *Ardeidae* and glossy ibis). Then, to simulate a typical Mediterranean hydro-system, they allow the site to slowly dry out, promoting a concentration of prey (e.g., fish and crustaceans), which in turn attracts waterbirds including spoonbills. This site is also relatively protected from anthropogenic disturbance, with only small groups of visitors guided by expert rangers being allowed into some sections of the area. This provides suitable conditions for newly fledged juveniles to forage safely with their parents, thus potentially explaining the high presence of spoonbills in the area during the early and late dispersal periods. Surprisingly, although immatures were present during early dispersal, they were absent during the late dispersal period, suggesting that there may be some competition for the area when resources are declining, so that the immatures could be displaced by the high number of adults and their young. By the end of August, the marshes used by spoonbills for foraging have largely dried up and spoonbills are absent from the area even during the wintering period, after managers have filled the marshes again with water in autumn. The persisting absence of spoonbills might be due to the relatively low abundance of fishes and crustaceans during all this period.

Recent studies suggest that the relation between protected and surrounding landscapes is complex, especially when considering highly mobile species that can move between these areas throughout the annual and life cycle (Nightingale et al. 2023; Soriano-Redondo et al. 2023). Our results suggest that privately managed areas play a complementary role in Camargue, where they are likely to provide suitable foraging areas for spoonbills and other waterbirds, through their management targeting waterfowl, when these are less available in strongly protected areas at certain periods of the year. However, considerable reliance on highly managed areas can have serious consequences for species dependent on them (Pernollet et al. 2015; Fasola et al. 2022; Champagnon et al. 2023). Indeed, changing land management practices may convert sites with suitable foraging resources into areas of low resource quality, unsuitable areas (e.g., abandonment of fish production; Mikuska et al. 2023). On the other hand, management such as that of the Vigueirat national nature reserve (see above), which provides high quality habitat while emulating local environmental conditions, may now be essential to maintain species resilience in a considerably modified and fragmented landscape. Thus, as is often recommended for bird species (Stephens et al. 2004), landscape-level approaches to habitat management are needed for the conservation of waterbirds (Qiu et al. 2024). By ensuring the presence of well-distributed foraging and roosting habitats across a highly heterogeneous landscape, it is possible to successfully mitigate the negative effects of disturbance and produce a more robust and resilient ecosystem (McDuie et al. 2021). Indeed, wetland ecosystems with heterogeneous landscapes promote waterbird diversity (Qiu et al. 2024). Nevertheless, understanding how specific management actions affect waterbird and habitat use is essential not only to enhance the effectiveness of conservation efforts, but also to promote wetland connectivity and species resilience in these fragmented landscapes.

## Supplementary Information

Below is the link to the electronic supplementary material.

Supplementary file1 (PDF 411 KB)

## Data Availability

The datasets generated during and/or analysed during the current study are available from the corresponding author on reasonable request.
