# Supplementary material for: Role of protected areas for a colonial-breeding waterbird in a fragmented landscape throughout its annual cycle
Source: Landsc Ecol. 2024 Dec 21;40(1):6. doi: 10.1007/s10980-024-02017-5 (PMC11828808; doi:10.1007/s10980-024-02017-5)
Supplement: Supplementary file 1 — Supplementary file1 (PDF 411 KB) [file 10980_2024_2017_MOESM1_ESM.pdf]

Supplementary Material

Title Journal name: Landscape Ecology

Manuscript type: Original Research paper

Manuscript Title: *Role of protected areas for a colonial-breeding waterbird in a fragmented landscape throughout its annual cycle*

Authors and affiliations: Hugo R. S. Ferreira[1,2*], José A. Alves[1,3], Frédéric Jiguet[4], Olivier Duriez[5], Thomas Blanchon[2], Tamar Lok[6], and Jocelyn Champagnon[2]

[1]Dep. Biologia & CESAM – Centre for Environmental and Marine Studies, Universidade de Aveiro, Campus de Santiago, 3810-193 Aveiro, Portugal.

[2]Tour du Valat, Research institute for the conservation of Mediterranean wetlands, Le Sambuc, 13200 Arles, France.

[3]South Iceland Research Centre, University of Iceland, Lindarbraut 4, 840-IS Laugarvatn, Iceland.

[4]Centre d'Ecologie et des Sciences de la Conservation, MNHN CNRS Sorbonne Université, M 43 Rue Buffon, CP 135, 75005, Paris, France

[5]CEFE, Univ Montpellier, CNRS, EPHE, IRD, 1919 route de Mende, Montpellier 34293, France

[6]NIOZ Royal Netherlands Institute for Sea Research, Department of Coastal Systems, PO Box 59, 1790 AB, Den Burg, Texel, The Netherlands

Corresponding Author: Hugo R. S. Ferreira (email: hr.ferreira@ua.pt)

Table S1. Summary of the spoonbills tagged in this study, identified by PCV code on the colour ring. Immature/Adult: age class at the time data used this study were obtained.

PVC ring	Date of GPS/GSM attachment	Mass before equipped (g)	GPS type	Attachment type	Age class when equipped	Immature/Adult data in Camargue
APVA	25/05/2016	2120	Skua-H 20g (Ecotone)	backpack harness	adult	No / Yes
ATTL	10/06/2016	1925	Skua-H 20g (Ecotone)	backpack harness	juvenile	Yes / Yes
ATZF	03/05/2017	1550	OrniTrack-25 3G (Ornitela)	backpack harness	adult	No / Yes
AXXX	07/05/2018	1700	OrniTrack-25 3G (Ornitela)	backpack harness	juvenile	Yes / Yes
AZZA	26/06/2018	1690	OrniTrack-25 3G (Ornitela)	backpack harness	juvenile	No / No
AZDC	17/04/2019	1640	OrniTrack-25 3G (Ornitela)	backpack harness	juvenile	No / No
AXVJ	13/06/2019	1840	OrniTrack-25 3G (Ornitela)	backpack harness	juvenile	No / No
AXVN	13/06/2019	1490	OrniTrack-25 3G (Ornitela)	backpack harness	juvenile	No / No
AXVP	13/06/2019	1350	OrniTrack-25 3G (Ornitela)	backpack harness	juvenile	No / No
AXVT	13/06/2019	1700	OrniTrack-25 3G (Ornitela)	backpack harness	juvenile	No / No
AXVV	13/06/2019	1520	OrniTrack-25 3G (Ornitela)	backpack harness	juvenile (1)	No / No
AXVZ	13/06/2019	1600	OrniTrack-25 3G (Ornitela)	backpack harness	juvenile	No / No
AZHS	13/06/2019	1450	OrniTrack-25 3G (Ornitela)	backpack harness	juvenile	No / No
AZZT	13/06/2019	1200	OrniTrack-25 3G (Ornitela)	backpack harness	juvenile (1)	No / No
AZZZ	13/06/2019	1550	OrniTrack-25 3G (Ornitela)	backpack harness	juvenile	No / No
FAFA	13/06/2019	1310	OrniTrack-25 3G (Ornitela)	backpack harness	juvenile	No / No
FANN	15/06/2020	1400	OrniTrack-25 3G (Ornitela)	backpack harness	juvenile	No / No
FBZA	09/07/2020	1425	Lego-LEG 25g (Interrex)	Tarsus Ring	juvenile	Yes / Yes
FBZB	09/07/2020	1525	Lego-LEG 25g (Interrex)	Tarsus Ring	juvenile	No / No
FBTZ	29/07/2020	1540	OrniTrack-25 3G (Ornitela)	backpack harness	juvenile	No / No
FBXA	29/07/2020	1560	OrniTrack-25 3G (Ornitela)	backpack harness	juvenile	Yes / No
FBXB	29/07/2020	1490	OrniTrack-25 3G (Ornitela)	backpack harness	juvenile	Yes / No
FBXC	29/07/2020	1625	OrniTrack-25 3G (Ornitela)	backpack harness	juvenile	No / No
FBXN	29/07/2020	1590	OrniTrack-25 3G (Ornitela)	backpack harness	juvenile	No / No
FBXX	29/07/2020	1650	OrniTrack-25 3G (Ornitela)	backpack harness	juvenile	No / No

A111	01/06/2021	1720	OrniTrack-25 3G (Ornitela)	backpack harness	juvenile	No / No
A112	01/06/2021	1410	OrniTrack-25 3G (Ornitela)	backpack harness	juvenile	No / No
A11A	01/06/2021	1500	OrniTrack-25 3G (Ornitela)	backpack harness	juvenile	No / No
A12A	29/07/2020	1700	OrniTrack-25 3G (Ornitela)	backpack harness	juvenile	No / No
A16T	01/06/2021	1300	OrniTrack-25 3G (Ornitela)	backpack harness	juvenile	No / No
FCAB	01/06/2021	1800	OrniTrack-25 3G (Ornitela)	backpack harness	juvenile	No / No
FCAF	01/06/2021	1750	OrniTrack-25 3G (Ornitela)	backpack harness	juvenile	No / No
FCAH	01/06/2021	1720	OrniTrack-25 3G (Ornitela)	backpack harness	juvenile	No / No
FCAT	01/06/2021	1700	OrniTrack-25 3G (Ornitela)	backpack harness	juvenile	Yes / Yes
A16J	08/06/2021	1520	OrniTrack-25 3G (Ornitela)	backpack harness	juvenile	No / No
A1NH	08/06/2021	1250	OrniTrack-25 3G (Ornitela)	backpack harness	juvenile	No / No
A2LA	16/06/2021	1700	OrniTrack-25 3G (Ornitela)	backpack harness	juvenile	No / No
A2LL	08/06/2021	1320	OrniTrack-25 3G (Ornitela)	backpack harness	juvenile	No / No
A2XC	08/06/2021	1400	OrniTrack-25 3G (Ornitela)	backpack harness	juvenile	No / No
A2XF	16/06/2021	1520	OrniTrack-25 3G (Ornitela)	backpack harness	juvenile	No / No
FCAN	08/06/2021	1690	OrniTrack-25 3G (Ornitela)	backpack harness	juvenile	No / No
FCCT	08/06/2021	1630	OrniTrack-25 3G (Ornitela)	backpack harness	juvenile	No / No
A116	01/06/2021	1580	OrniTrack-25 3G (Ornitela)	backpack harness	juvenile	Yes / No
A16C	01/06/2021	1440	OrniTrack-25 3G (Ornitela)	backpack harness	juvenile	No / No
A16L	01/06/2021	1670	OrniTrack-25 3G (Ornitela)	backpack harness	juvenile	No / No
A16P	01/06/2021	1500	OrniTrack-25 3G (Ornitela)	backpack harness	juvenile	No / No
A1NJ	08/06/2021	1600	OrniTrack-25 3G (Ornitela)	backpack harness	juvenile	No / No
A1NP	16/06/2021	1450	OrniTrack-25 3G (Ornitela)	backpack harness	juvenile	Yes / Yes
A1TH	01/06/2021	1500	OrniTrack-25 3G (Ornitela)	backpack harness	juvenile	Yes / Yes
A2A1	16/06/2021	1590	OrniTrack-25 3G (Ornitela)	backpack harness	juvenile	No / No
A2X2	16/06/2021	1550	OrniTrack-25 3G (Ornitela)	backpack harness	juvenile	No / No
A2XZ	16/06/2021	1820	OrniTrack-25 3G (Ornitela)	backpack harness	juvenile	No / No
FCAD	01/06/2021	1250	OrniTrack-25 3G (Ornitela)	backpack harness	juvenile	Yes / No
FCAJ	01/06/2021	1340	OrniTrack-25 3G (Ornitela)	backpack harness	juvenile	No / No
FCCS	01/06/2021	NA	OrniTrack-25 3G (Ornitela)	backpack harness	juvenile	No / No

A2NL	08/06/2021	1440	OrniTrack-25 3G (Ornitela)	backpack harness	juvenile	No / No
A2X3	08/06/2021	1400	OrniTrack-25 3G (Ornitela)	backpack harness	juvenile	No / No
A2DB	16/06/2021	1620	OrniTrack-25 3G (Ornitela)	backpack harness	juvenile	No / No
A2DD	16/06/2021	1600	OrniTrack-25 3G (Ornitela)	backpack harness	juvenile	Yes / Yes
A2L1	16/06/2021	1570	OrniTrack-25 3G (Ornitela)	backpack harness	juvenile	No / No
A2L6	16/06/2021	1700	OrniTrack-25 3G (Ornitela)	backpack harness	juvenile	No / No
A2L9	16/06/2021	1480	OrniTrack-25 3G (Ornitela)	backpack harness	juvenile	No / No
A2NS	16/06/2021	1500	OrniTrack-25 3G (Ornitela)	backpack harness	juvenile	No / No
A1J6	26/04/2022	1580	OrniTrack-25 3G (Ornitela)	backpack harness	juvenile	Yes / No
A1J9	26/04/2022	1540	OrniTrack-25 3G (Ornitela)	backpack harness	juvenile	No / No
A1AZ	02/06/2022	1610	OrniTrack-25 3G (Ornitela)	backpack harness	juvenile (1)	No / No
A23A	02/06/2022	1780	OrniTrack-25 3G (Ornitela)	backpack harness	juvenile	No / No
A23B	02/06/2022	1760	OrniTrack-25 3G (Ornitela)	backpack harness	juvenile	Yes / No
A212	08/06/2022	1780	OrniTrack-25 3G (Ornitela)	backpack harness	juvenile	No / No
A25D	08/06/2022	1640	OrniTrack-25 3G (Ornitela)	backpack harness	juvenile	No / No
A251	20/06/2022	1780	OrniTrack-25 3G (Ornitela)	backpack harness	juvenile	No / No
A252	20/06/2022	1600	OrniTrack-25 3G (Ornitela)	backpack harness	juvenile	Yes / No
A255	20/06/2022	1380	OrniTrack-25 3G (Ornitela)	backpack harness	juvenile	No / No
A22B	26/04/2023	2000	OrniTrack-25 3G (Ornitela)	backpack harness	juvenile	Yes / No
A22C	26/04/2023	1800	OrniTrack-25 3G (Ornitela)	backpack harness	juvenile	No / No
A22J	26/04/2023	1900	OrniTrack-25 3G (Ornitela)	backpack harness	juvenile	No / No
A22N	26/04/2023	1700	OrniTrack-25 3G (Ornitela)	backpack harness	juvenile	No / No
A22D	23/05/2023	1850	OrniTrack-25 3G (Ornitela)	backpack harness	juvenile	No / No
A22F	23/05/2023	1900	OrniTrack-25 3G (Ornitela)	backpack harness	juvenile	No / No
A22H	23/05/2023	2000	OrniTrack-25 3G (Ornitela)	backpack harness	juvenile	No / No
A22P	23/05/2023	1900	OrniTrack-25 3G (Ornitela)	backpack harness	juvenile	Yes / No
A22S	23/05/2023	1850	OrniTrack-25 3G (Ornitela)	backpack harness	juvenile	No / No
A3AC	23/05/2023	1980	OrniTrack-25 3G (Ornitela)	backpack harness	juvenile	No / No
A3AF	23/05/2023	1550	OrniTrack-25 3G (Ornitela)	backpack harness	juvenile	No / No
A3AT	23/05/2023	1950	OrniTrack-25 3G (Ornitela)	backpack harness	juvenile	No / No

A2ZP	25/05/2023	1810	OrniTrack-25 3G (Ornitela)	backpack harness	juvenile	No / No
A235	25/05/2023	1820	OrniTrack-25 3G (Ornitela)	backpack harness	juvenile [(1)]	No / No
A3CN	25/05/2023	1790	OrniTrack-25 3G (Ornitela)	backpack harness	juvenile	No / No
A3C1	25/05/2023	1820	OrniTrack-25 3G (Ornitela)	backpack harness	juvenile [(1)]	No / No
A3C2	25/05/2023	1580	OrniTrack-25 3G (Ornitela)	backpack harness	juvenile	Yes / No
A3C6	25/05/2023	1810	OrniTrack-25 3G (Ornitela)	backpack harness	juvenile	No / No
A3AH	15/06/2023	1620	OrniTrack-25 3G (Ornitela)	backpack harness	juvenile	Yes / No
A3AJ	15/06/2023	1560	OrniTrack-25 3G (Ornitela)	backpack harness	juvenile	Yes / No
A5AX	15/06/2023	1680	OrniTrack-25 3G (Ornitela)	backpack harness	juvenile	No / No
FCNZ	15/06/2023	1720	OrniTrack-25 3G (Ornitela)	backpack harness	juvenile	No / No
FDDT	15/06/2023	1650	OrniTrack-25 3G (Ornitela)	backpack harness	juvenile	No / No

note [(1)]: No valid fixes in Camargue even as juveniles, individuals completely excluded from the analysis.

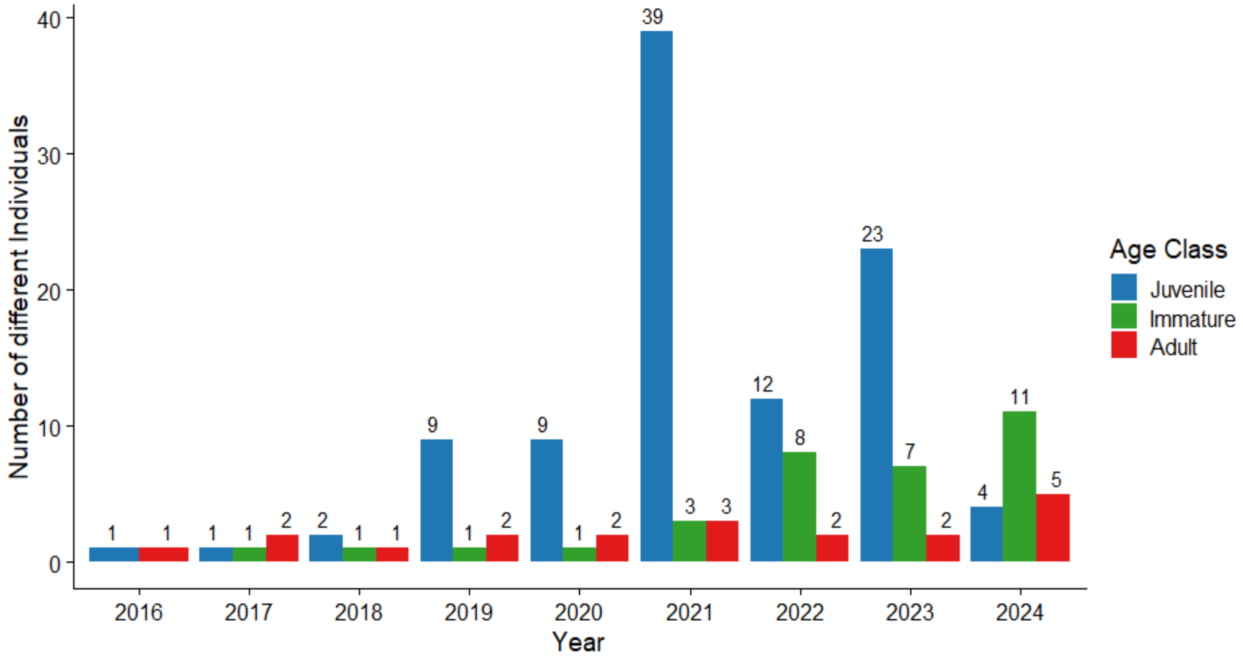

Fig. S1 – Summary of number of different spoonbills with valid revisitation fixes (for this study) in Camargue per year and per age class.

In the years of 2019 and 2021, an egg exchange with the Netherlands occurred, meaning that some individuals in our study have different genetics from the rest of the population (Dutch origins: $n_{2019} = 6$ | $n_{2021} = 15$). However, there is no *a priori* reason to believe that the genetics of these birds could influence their presence in strongly protected areas or not. Indeed, when comparing birds with Dutch origin against French birds, we did not detect any significant difference in the number of fixes within strongly protected areas, and thus, all individuals were kept in the analysis (Table S2).

Table S2. Summary of a general linear mixed model analysis of individual genetic origin (Dutch and French) and land protection levels (moderate and strong), amount of revisitation fixes. Interaction between genetic origin and land protection levels, was also considered.

**Coefficient**	**Estimates**	**Standard error**	***z*-value**	***Pr(>|z|)***	***p*-value**	***2.5%***	***97.5%***
(Intercept)	7.9	0.2	40.9	0.0	***	7.6	8.3
Dutch	0.3	0.3	0.7	0.5		-0.3	0.7
Strong	-0.5	0.1	-4.6	0.0	***	-0.7	0.0
Dutch × Strong	0.0	0.3	-0.1	0.9		-0.6	0.5

*Note: 0 '***' 0.001 '**' 0.01 '*' 0.05 '.' 0.1 ' ' 1*

For the sensitivity analysis we adapted the methodology described by Rodrigues et al. (2023). Due to the amount and complexity of the data (i.e. multiple individuals, across multiple years, age classes, and different ecological periods), as well as a lack of computing power, we only performed this analysis on a subset of 20 random individuals (ca. 23% of the individuals). First, we calculated the average number of revisits to all areas per radius value and observed that, on average, there is a minimal increase in the mean number of revisits after a radius of 100m despite a higher chance of overlapping radii. Additionally, we plotted the variance of the log of revisits

across the same radii and at 100m it appears to contain a considerable amount of the population variance (Fig. S2). Finally, we performed a sensitivity analysis to confirm that changing the selected radius by -50 to +150 metres would not impact the percentage of fixes in strongly protected areas (Fig. S3 and Table S3). In conclusion, these plots suggest that a 100-meter radius is a suitable radius choice as it captures a significant number of revisits while also providing a high degree of variance (Fig. S2) and not significantly changing the percentage of strongly protected areas compared to other radii (Fig. S3 and Table S2).

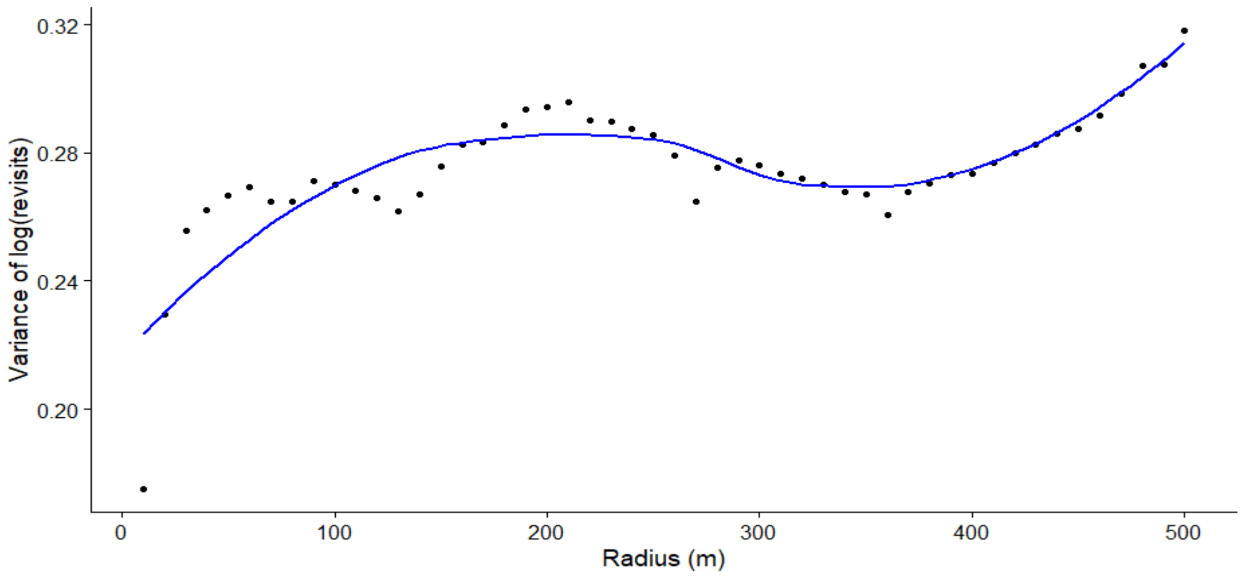

Fig. S2 – Variation on the log of revisits made by the Camargue population per radius tested. Blue line represents the LOESS curve that smooths the scatter plot points to reveal the trend in variance as the radius increases.

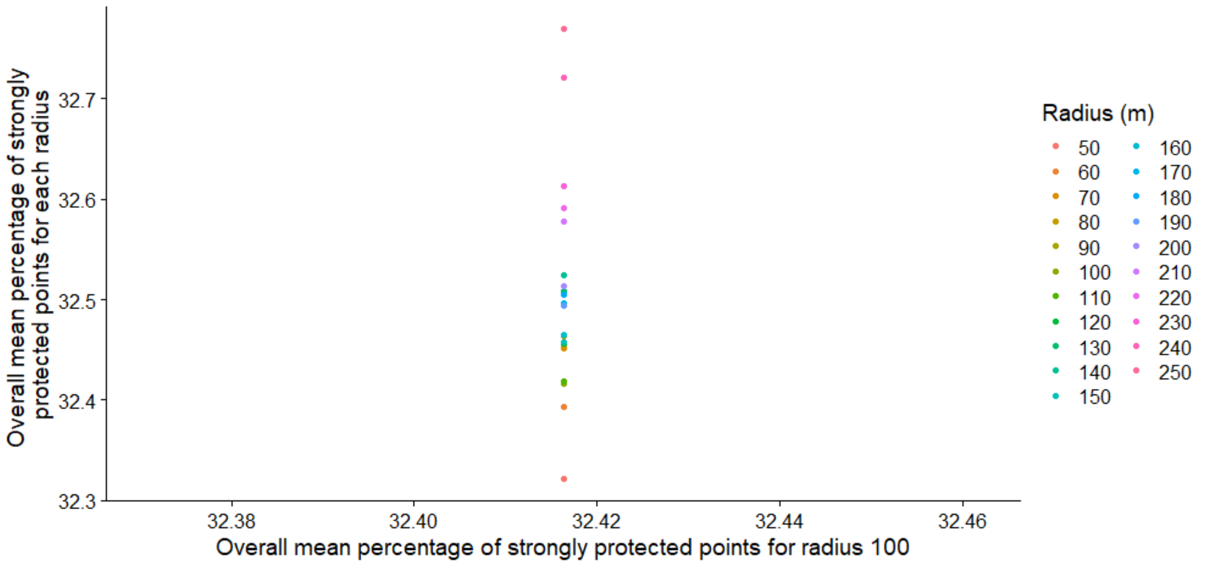

Fig. S3 – Variation in the mean percentage of fixes within strongly protected areas while varying the values of the radius in the revisitation analysis. See Table S3 to see the lack of statistical significance between radius results.

Table S3 – The R- and p-values show the level of association between the percentage of fixes within strongly protected areas using a radius of 100 m and several other proposed radii (from -50 to +150m), in the revisitation analysis.

Radius	R	*p*-value	Radius	R	*p*-value
50	32.32	0.988	160	32.46	0.994
60	32.39	0.997	170	32.50	0.990
70	32.45	0.995	180	32.51	0.988
80	32.46	0.994	190	32.49	0.990
90	32.45	0.995	200	32.51	0.987
110	32.42	1.000	210	32.58	0.979
120	32.46	0.995	220	32.59	0.977
130	32.51	0.988	230	32.61	0.975
140	32.52	0.986	240	32.72	0.961
150	32.46	0.995	250	32.77	0.954

Table S4. Total number of revisitation fixes considered per combination of age class and period of the annual cycle ($n_{Revisitation\ fixes}$= 1,565,010; n_{Ind}= 91).

Age class × Period	Total number of revisitation fixes
**Juvenile × Breeding**	-
**Juvenile × Early dispersal**	473898
**Juvenile × Late dispersal**	441721
**Juvenile × Wintering**	69399
**Immature × Breeding**	187183
**Immature × Early dispersal**	181171
**Immature × Late dispersal**	90102
**Immature × Wintering**	36402
**Adult × Breeding**	50186
**Adult × Early dispersal**	27227
**Adult × Late dispersal**	6607
**Adult × Wintering**	1114

Table S5. Summary of a general linear mixed model analysis of age class (juvenile, immature, and adult), land protection levels (moderate and strong), and period (breeding, early dispersal, late dispersal, and wintering) in amount of revisitation fixes. The interaction between age class and land protection levels, as well as the one between land protection levels and period, were also included.

**Coefficient**	**Estimates**	**Standard error**	***z*-value**	**Pr(>|z|)**	***p*-value**	***2.5%***	***97.5%***
(Intercept)	8.8	0.3	29.7	0.0	***	8.2	9.4
Strong	-2.0	0.4	-5.5	0.0	***	-2.7	-1.3
Early dispersal	-0.6	0.2	-2.6	0.0	**	-1.1	-0.2
Late dispersal	-1.1	0.3	-4.2	0.0	***	-1.6	-0.6
Wintering	-1.6	0.3	-5.9	0.0	***	-2.2	-1.1
Immature	-0.3	0.2	-1.6	0.1		-0.7	0.1
Adult	-1.2	0.3	-4.4	0.0	***	-1.7	-0.7
Strong × Early dispersal	1.4	0.3	4.1	0.0	***	0.7	2.1
Strong × Late dispersal	1.9	0.4	5.1	0.0	***	1.2	2.6
Strong × Wintering	1.7	0.4	4.2	0.0	***	0.9	2.5
Strong × Immature	-0.3	0.2	-1.1	0.3		-0.8	0.2
Strong × Adult	0.6	0.3	1.9	0.1	.	0.0	1.2

*Note: 0 '***' 0.001 '**' 0.01 '*' 0.05 '.' 0.1 ' ' 1*

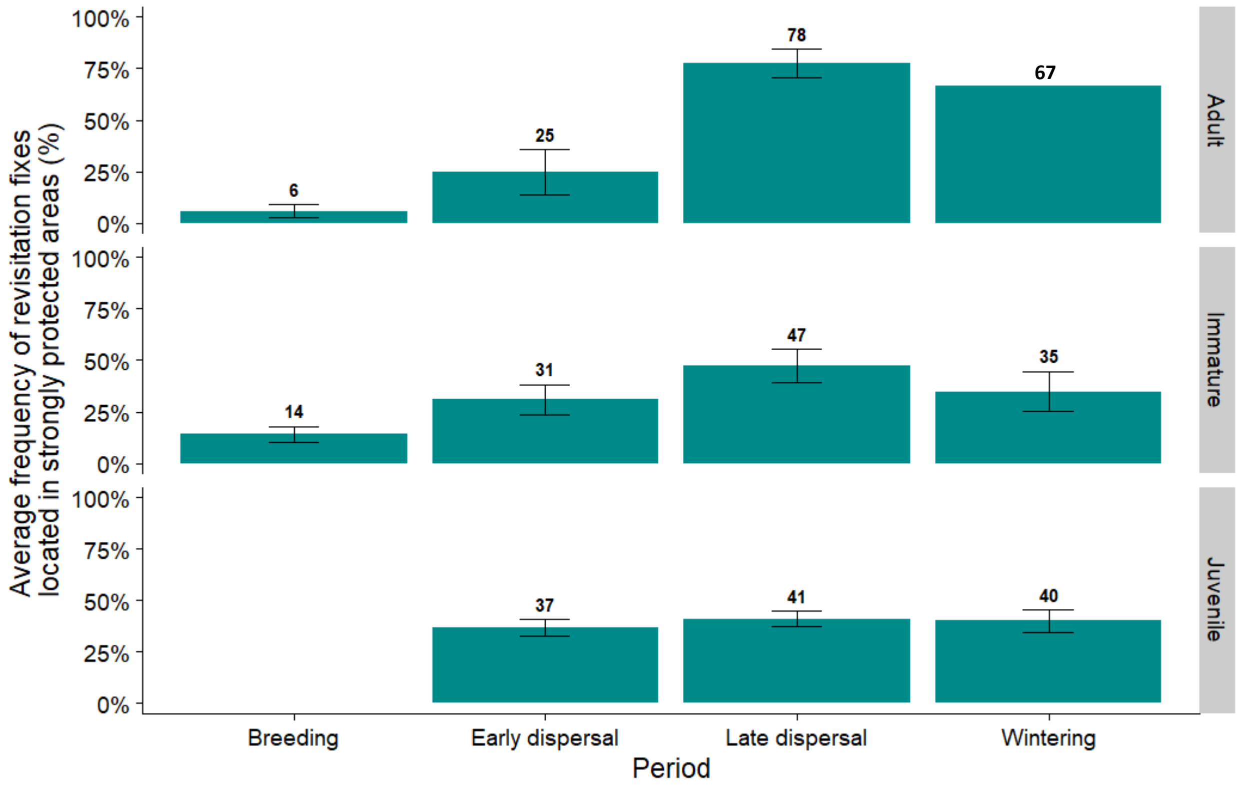

Fig. S4 – Variation in the average frequency of revisitation fixes ($n_{Revisitation\ fixes}$= 1,565,010; n_{Ind}= 91), throughout the annual cycle (breeding, early dispersal, late dispersal, and wintering) according to age class (juvenile, immature, and adult). Whiskers indicate the standard error around the average proportion of each group with averaged frequency of revisitation fixes provided above them.

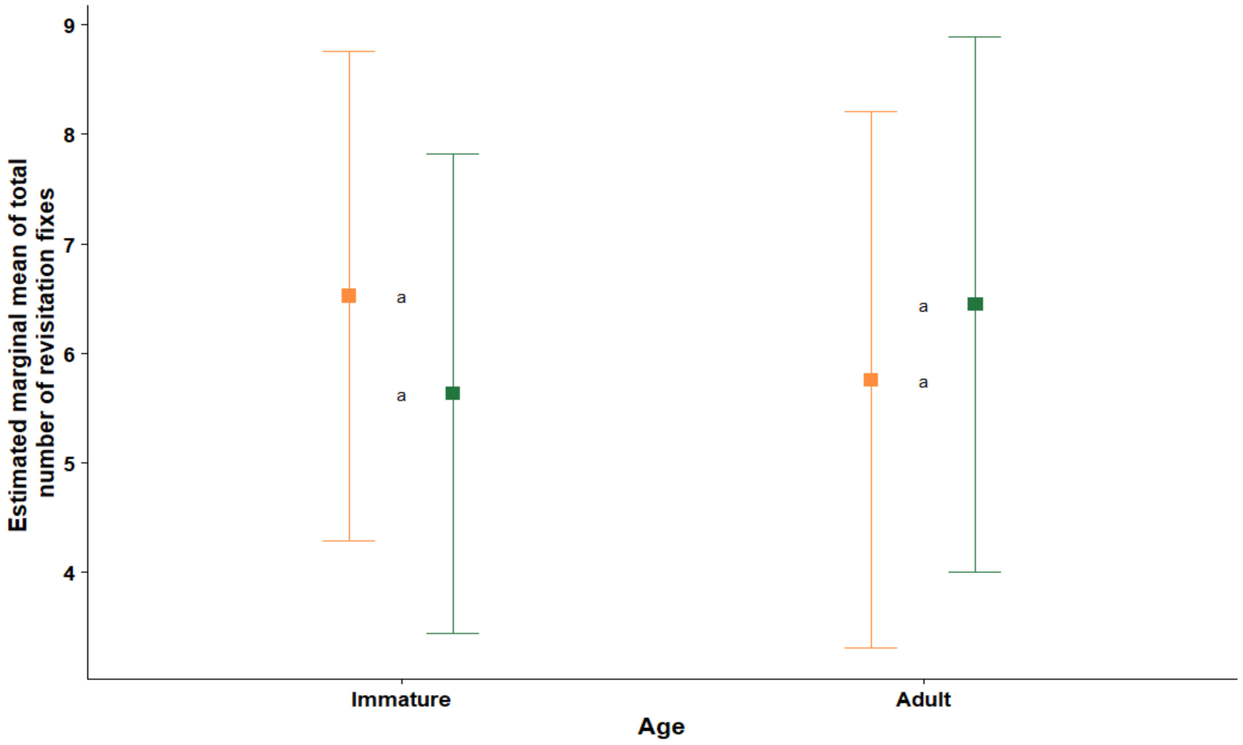

Fig. S5 – Post-hoc comparisons of estimated marginal mean of total revisitation fixes between protection (moderate – orange; strong – green) across the different age class (immature; and adult) during the wintering period. Vertical lines indicate 95% confidence interval after Sidak correction. Different letters indicate statistical differences between groups ($p < 0.05$).
